# 15 years supporting adherence to oral anti-cancer treatment: use of the MASCC Oral Agent Teaching Tool (MOATT) worldwide, a review for the future

**DOI:** 10.1007/s00520-025-09274-3

**Published:** 2025-02-27

**Authors:** Sultan Kav, Mapi Fleury, Paz Fernández-Ortega, Ellen F. Manzullo, Kimberley-Ann Kerr, Regina DeGennaro, Pamela K. Ginex

**Affiliations:** 1 Faculty of Health Sciences Department of Nursing, Baskent University, Ankara, Turkey; 2 Department of Oncology, Faculty of Biology and Medicine, Lausanne University Hospital (CHUV), Lausanne, Switzerland; 3 Department of Nursing Research, Catalan Institute of Oncology, Hospital Duran I Reynals, Barcelona, Spain; 4 Department of General Internal Medicine, The University of Texas MD Anderson Cancer Center, Houston, TX USA; 5 SA Health, Adelaide, South Australia Australia; 6 School of Nursing Charlottesville, University of Virginia, Charlottesville, VA USA; 7 School of Nursing, Stony Brook University School of Nursing, Stony Brook, NY USA

**Keywords:** MASCC oral agent teaching tool, Therapeutic adherence, MOATT, Implementation

## Abstract

**Introduction:**

The MASCC Oral Agent Teaching Tool (MOATT®) was developed to address the challenges of patient education and adherence in the context of oral anticancer agents. Despite its evidence-based design and global availability, there is limited documentation on its application in clinical practice and research. This review aims to assess the extent of MOATT usage and evaluate the impact on patient outcomes.

**Methods:**

A scoping review was conducted following Joanna Briggs Institute and PRISMA-ScR reporting standards. Databases included CINAHL, Embase, PsycInfo, Web of Science, and PubMed. Inclusion criteria were studies published between 2010 and 2023 that used MOATT in clinical practice or research. Two reviewers independently screened and extracted data, with discrepancies resolved by consensus.

**Findings:**

Seventeen studies met the inclusion criteria, from nine countries. The MOATT was most used by nurses and pharmacists to guide patient education when initiating new oral anticancer therapies. Reported outcomes included improved patient knowledge, understanding of medication regimens, and comfort in managing treatments. However, only six studies assessed medication adherence, with mixed results. The tool was adapted in various settings, yet there was a notable lack of detailed reports on its usage and outcomes, highlighting underutilization and potential barriers to broader implementation.

**Conclusion:**

The MOATT is a valuable tool for supporting patients on oral anticancer agents yet is underutilized in practice. Future research should focus on understanding the barriers to its adoption, exploring patient and provider perspectives, and integrating implementation science to enhance its use in diverse clinical contexts.

## Introduction

No less than 106 oral antineoplastic agents are listed in the 2023 Multinational Association of Supportive Care in Cancer (MASCC)/European Society of Medical Oncology (ESMO) classification of the emetogenic potential of anticancer drugs [1], a staggering number that does not even include the anti-hormonal medications and other agents that accompany anti-cancer treatments and must be taken daily. The transition to oral therapies over the past few decades has significantly transformed the cancer care delivery model. While this shift has brought new opportunities for patient-centered care, it has also introduced significant challenges for oncology professionals.

Specifically, ensuring that patients are adequately supported to manage their treatment at home has become increasingly complex. There is now a growing expectation that patients and caregivers will assume a range of intricate tasks, including the administration of medication, self-management of symptoms and side effects, monitoring for drug interactions, and adherence to treatment [2]. Despite the critical importance of adherence, studies on oral chemotherapy reveal a troubling range in compliance levels, from 46 to 100% [3]. The oncology care team plays a pivotal role in this regard, providing education and follow-up in a comprehensive and consistent manner to support adherence to therapy [4]. From this perspective, evidence-based recommendations include the use of adherence risk assessment, adherence education, ongoing assessment, proactive follow-up, coaching, and motivational interviewing, in addition to usual care [5]. In this context, it is imperative that all members of the medical oncology team, including nurses, doctors, and pharmacists, seize the opportunity to educate patients on the aims of treatment, the correct administration of drugs, and the management of side effects [6].

In light of the a need for education regarding medication adherence and safety, along with the absence of standardized teaching strategies, the Multinational Association of Supportive Care in Cancer developed the MASCC Oral Agent Teaching Tool (MOATT®) to assist healthcare providers in assessing and teaching patients undergoing oral cancer treatment [7, 8]. The implementation of an intervention such as the MOATT at the point of care is contingent upon several variables. The suitability of the intervention for clinical staff and patients, as well as the implementation strategies employed, influence the extent to which the intervention is transferred to practice. For the MOATT to be effective and sustainable, it must not only meet the specific needs of the target population, but also consider the particularities of the local context. MOATT has been disseminated and promoted by MASCC for more than a decade. With the rapidly changing cancer treatment landscape, it was necessary to assess whether the MOATT's objectives remained aligned with the evolving needs and contexts of oncology healthcare providers and patients. This paper presents a brief historical overview of the MOATT, its current format, a review of published papers on its reported uses in clinical practice and research, and avenues for reflection and future research.

## Development of the MOATT

The construction of the MOATT was a multinational and interprofessional effort that began in 2005, with the international extension of a Turkish team’s study that revealed a dearth of support for patients undergoing oral anticancer treatments [9]. Following a comprehensive literature review and group work to identify the elements of the tool, the core components were subjected to an evaluation by a pharmacist and nurses to ensure their comprehensiveness, accuracy, and cultural sensitivity [8]. In June 2008, nurse coordinators and pharmacists representing 15 countries were invited to attend a “Train the Trainer” workshop. Following the workshop, clinical implementation, and evaluations of the MOATT were conducted involving 635 patients and caregivers by 114 nurses in China, Denmark, Greece, Kenya, Spain, Turkiye, and the United States. The initial version (1.0) of the MOATT has been translated into 14 languages with the objective of facilitating the expeditious adoption and dissemination of a research and clinical practice instrument.

MOATT has consistently been regarded as an instrument designed to benefit both patients and oncology healthcare providers. However, its underutilization was identified in 2012 [7], prompting an initial review for an update and the subsequent creation of a user guide for healthcare professionals to assist with the application of the method in clinical and research settings [10]. The tool (1.2) is now available in nine languages in a certified translation [11].

The original objective of the MOATT was to develop a standardized teaching methodology. The MOATT provides a structured format to ensure that all key areas of patient assessment and teaching are addressed. The tool contains four sections; the first lists key questions to assess the patient's knowledge of the treatment plan, current medications, and ability to obtain and take an oral cancer agent. The second section contains general patient teaching instructions applicable to all oral cancer agents, such as storage, handling, and disposal, identifying a system for remembering to take the drug, and actions to take for various situations, such as a missed dose. The third section is used to provide drug-specific information, such as dose and schedule, side effects, and potential interactions. The last section lists questions that may be asked to ascertain understanding of the information provided. There is an additional page as a handout of drug-specific information that can be provided to the patient in the absence of any other prepared information.

## Scoping review method

The objective of this scoping review is to identify published reports on the utilization of the MOATT in clinical care and research. This study aims to describe the manner and context in which the MOATT is being utilized, identify strategies and barriers to its implementation, and evaluate adherence and patient outcomes associated with its use. Additionally, the authors sought to identify potential areas for updating the MOATT, if necessary, based on findings from the literature.

## Design

This scoping review [12–15] was conducted following established Joanna Briggs Institute scoping review guidelines [16]. Reporting follows the Preferred Reporting Items for Systematic Reviews and Meta-Analyses (PRISMA) Extension for Scoping Reviews [13, 17]. Consistent with the purpose of a scoping review, the focus was to achieve a high-level overview of the evidence.

## Search strategy and selection criteria

A clinical librarian developed the search strategy in collaboration with the research team. Searches in CINAHL, Embase, PsycInfo, Web of Science, and PubMed were conducted with the following search terms: “MASCC Oral Agent Teaching Tool” or “MOATT.” Inclusion criteria included a cancer population, use of the MOATT in clinical practice or research, published 2010-present (after the MOATT was published), and any language. We cross-referenced the list of projects/research on the MASCC website and additional articles/abstracts submitted by team members for a total of 57 articles identified. Articles that only mentioned the MOATT for theoretical or discussion purposes but did not use it in planning or conducting a study or in clinical practice were excluded.

## Study selection and data extraction

Two reviewers independently, and in duplicate, screened titles and abstracts to assess eligibility. Papers meeting eligibility criteria progressed to full-text review, which was also conducted independently and in duplicate. Disagreements were discussed between reviewers at each phase of the process, and if consensus was not reached, a third reviewer was consulted.

Reviewers independently extracted data from each published report into a standardized and pilot-tested spreadsheet. Extracted data included: article and author demographics, type of article, patient population, setting, how the MOATT was utilized, and medication adherence outcomes. After extracting the data, a second reviewer independently reviewed the information, and discrepancies were resolved by consensus.

## Findings

### Study characteristics

**Fig. 1:**
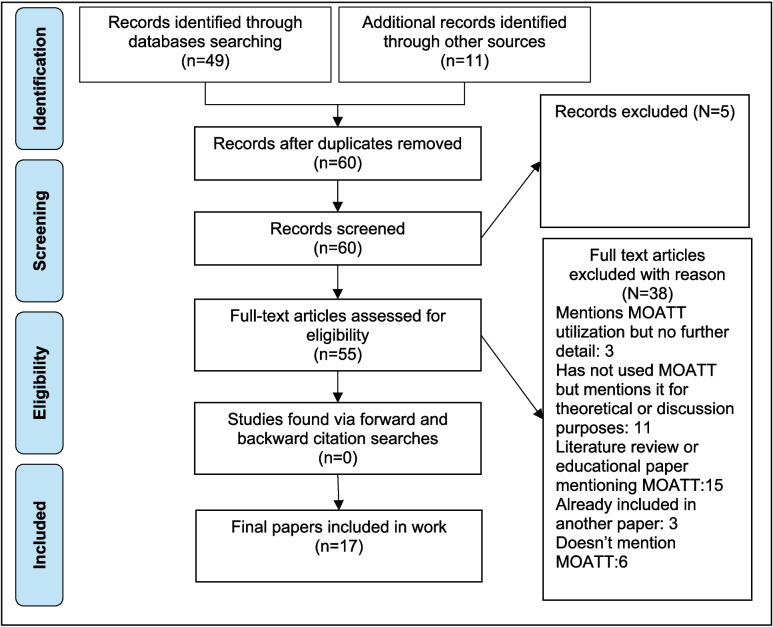
PRISMA flowchart

Figure 1 presents a flow chart illustrating the progression of papers through the review stages. Table 1 provides a summary of the characteristics of the reports.

**Table 1:** Study characteristics summary

Author, Year & Country	Population & Sample Size	Oral anti-cancer agents	Use of MOATT				Outcomes & Comments
			Initial Teaching	Follow-up	Full (F) Part (P)	Translation	
Campbell, 2014; Canada [18]	Mixed, 20	Mixed	✓	✓	P		Increased knowledge; decrease in grade 3/4 adverse events
Boucher et al. 2015; USA [19]	Lung cancer, 29	Erlotinib	✓	✓	F		Resulted in high scores for patient knowledge and adherence
Bellomo, 2016; USA [20]	Mixed, 24	Oral CT agents	✓		F		The patient-centered assessment, education protocol & the tailored nurse follow-up telephone call protocol effective in promoting symptom management & adherence. Become a standard of practice for oral chemotherapy patients at the Hospital
Tokdemir & Kav, 2017; Turkiye [21]	Mixed; 41	Mixed	✓	✓	F	Turkish	Individual education with the MOATT and follow‐up for patients receiving oral agents for cancer treatment increased patient medication adherence self‐efficacy
Riese et al. 2017; Germany [22]	Mixed; 165	Mixed	✓	✓	F		The patients of the intervention group reported fewer side effects (skin rash, pain, fatigue, nausea, vomiting) and reduced unplanned therapy interruptions
Byrne et al. 2018; Australia [23]	Mixed; 29	Mixed	✓		F		Improved patient understanding; pharmacist-led OAM management clinic was a valuable service
Newman, 2018; USA [24]	Mixed, 10	Mixed	✓	✓	F		Improved medication adherence; fostered and improved healthcare provider relationships; supporting & increasing the use of positive self care strategies
Roberts et al. 2018; USA [25]	Mixed; 31	Mixed	✓	✓	F		Improvement in safe handling/ storage, drug-drug and food interactions, and plan for missed doses
He et al. 2019; China [26]	NSLC; 44	Gefitinib	✓	✓	F		MOATT was not only beneficial to the patients in terms of QoL and psychological wellbeing, but also effective for reducing side effects
Hartwell et al. 2019; USA [27]	Mixed; 56	Mixed	✓		F		Safe handling/storage, drug-drug and food-drug interactions and plan for missed doses all improved
Tadic et al. 2020; Serbia [28]	Breast; 142	Capecitabine	✓		F	Serbian	Depression, anxiety and stress decreased significantly in the experimental group
Dürr et al. 2021; Germany [29]	Mixed, 202	Mixed	✓	✓	F		Intensified clinical pharmacological/ pharmaceutical care has considerable effects on the number of medications errors (−34%), patient treatment satisfaction & severe side effects (−45%)
Tolotti et al. 2021; Switzerland [30]	Mixed; 142	Mixed		✓	F	Italian	The survey questionnaire & interview questions were prepared based on MOATT to understand adherence. Overall, patients were satisfied with the education
Lin et al. 2021; Germany [31]	Mixed;58	Oral CT agents	✓	✓	F		Most participants found the intervention to be very beneficial. MOATT adapted with awareness of potentially lower health literacy and language barriers
Gallagher 2021; USA [32]	Multiple myeloma;11	Oral CT agents	✓	✓	F		Decrease in ASK-12 medication adherence scores from pre (M = 18.64, SD = 3.85) to post (M = 18.27, SD = 3.66). Although the change in medication adherence was not statistically significant, the intervention decreased barriers & problems with adherence
Wang et al. 2022; China [33]	Lung cancer; 95	Mixed	✓	✓	P		Adherence and QoL increased in the intervention group
Fariya et al. 2022; India [34]	Mixed; 186	Mixed	✓	✓	P		Patients had initial teaching followed by follow up telephone calls

A total of 17 articles were identified that used the MOATT in clinical practice or research, originating from nine countries. Six of these were from the United States, three from Germany, two from China, and the remaining countries contributed a single study each. The remaining countries were Australia, Canada, India, Serbia, Switzerland, and Turkiye. Fifteen of the reports were research studies (all quantitative with two mixed methods studies), and two were quality improvement projects. All reports were conducted on a general adult population, with one study focusing only on older adults. Most papers included a mixed sample of cancer diagnoses (*n* = 12), with three focused on lung cancer, one on breast cancer, and one sample included patients with multiple myeloma. Sample sizes ranged from 11 to 202 in the eligible articles. Many of the articles included patients receiving a combination of oral anticancer agents (*n* = 14), with three studies examining the use of a specific oral anticancer agent (erlotinib, gefitinib, and capecitabine). Translated versions of the MOATT were used in the studies as Turkish [21, 35], Serbian [28], and Italian [30].

### Use of MOATT

MOATT was used in a variety of ways in the identified articles. Most often, the MOATT was used to guide initial education from oncology healthcare providers (nurses and pharmacists) for patients starting a new oral anti-cancer agent. For example, one study used the MOATT to guide initial teaching by pharmacists and when asked to ‘teach back’ the education, patients exhibited consistent reports of understanding, particularly regarding the oral anti-cancer medication's name, dosing schedule, storage and handling instructions [31].

Six studies assessed adherence with validated measures, the ASK-12 [20, 24, 32], Morisky Medication Adherence Scale (MMAS-8) [19, 33], and the Medication Adherence Self-Efficacy Scale (MASES) [21]. These studies reported improved knowledge and understanding of medication regimens [19, 20, 32] and increased patient comfort managing their treatments [20] and that the MOATT was feasible to use in practice [19]. One study [21] found an increase in medication adherence self-efficacy with the MASES (66.39 vs 71.04; *p* < 0.05). Other outcomes studied, including treatment satisfaction [29], knowledge and understanding of treatment [18, 19, 23], reduction of side effects [22, 26, 29] and safe handling and storage of medication [25, 27], all showed an improvement from pre to post intervention.

One study used the MOATT to guide their questionnaire and semi-structured interview development to describe the patient and nurse perception of the effectiveness of education in oral cancer treatment [30]. Use of the MOATT to guide the study was successful, with the study finding high levels of patient satisfaction with the education received. Of the studies reviewed, none described implementation strategies or barriers to using the MOATT.

## Discussion

Supporting people who are taking oral anti-cancer agents is a priority for the oncology clinical team. The MOATT was initially developed to meet the need for evidence-based standardization of training in the field of therapeutic adherence to oral therapies. As oncology care has changed rapidly over the past decade, a review of how the MOATT was being used in reported studies was deemed appropriate.

In this scoping review, we aimed to describe the contexts in published literature in which the MOATT has been used, the advantages and disadvantages for the patient or the healthcare professionals, as well as to identify needs for a potential update. Given the limited number of articles included in the study (*n* = 17), despite being evidence-based and available as part of a supportive care approach, the MOATT is being underutilized for research and clinical care.

Studies that have utilized the MOATT have reported positive results including increased knowledge, reduction in side effects, improvement in safe handling and adherence to oral anti-cancer medications [18–22, 24, 26, 29, 33]. Several studies used the MOATT to guide the development of their intervention and did not use the full MOATT [18, 33, 34].

Given the abundance of reviews and clinical practice guidelines that recommend MOATT, the limited reports of its use in the literature warrant reflection. Our literature search identified studies that discussed the MOATT but did not report using it in practice [36–38]. A significant question remains unanswered: why have medical teams who reference MOATT in their discussions or theoretical work not used it in clinical care or research? In some instances, the MOATT has been described as a potentially beneficial tool that could be employed, but unfortunately, was not utilized [37]. This raises an important question: What are the underlying factors that contribute to the underutilization of MOATT?

One factor may be the general challenge of changing practice and implementing a new intervention at the point of care. The field of evidence-based practice and implementation science has identified barriers and facilitators to implementing practice change. To be effective and sustainable, interventions must meet the specific needs of the end users and consider the particularities of the local context. A first step to implementation entails identifying factors to be considered at different levels (individual, organizational, patient, provider, system, etc.), the factors influencing implementation (facilitators, obstacles), and the stakeholders (clinical, staff, patients and caregivers) impacted by this practice change. Once the determinants have been identified, the next step is to select one or more strategies for implementing an intervention that considers the local needs and context. The articles identified in this scoping review did not report implementation strategies or barriers. Without this knowledge, practice change will remain a challenge. It is important that future research on implementation of practice change interventions to support medication adherence include implementation details.

In addition to details on implementation of the MOATT, there are other opportunities that should be considered for a future revision. Kunitomo and colleagues [39] used focus groups of nurses to identify barriers to implementation of the MOATT. Barriers identified included system level (staffing and multidisciplinary collaboration) and patient-level (lack of educational strategy such as goal setting) and that patient groups, such as older adults, may need individualize interventions [39]. Other elements of the MOATT that could be subjected to revision include the emphasis on knowledge, limited consideration of patients’ beliefs, and a lack of suitability as a tool for measuring adherence. An implementation guide that includes an update of the case studies and research application with a focus on implementation would be beneficial to future clinicians and researchers. The guide should contain recommended outcome measures to enable synthesis of results across multiple studies, including a measure of medication adherence.

The MOATT has many strong components that should be maintained. It has been translated into nine languages and is likely being used in more clinical situations than is reported in the literature. The MOATT guide includes information on its development as well as case studies of use in practice and application in research. Each of these allows the end user to have confidence in its development and use case studies to guide use at clinical sites.

The limited references to MOATT in the literature should not be taken as an indication that the tool has not been beneficial to oncology healthcare professionals. An analysis of the MOATT website's data reveals that from January 2023 to August 2024, this page (https://mascc.org/resources/assessment-tools/mascc-oral-agent-teaching-tool-moatt/) has been accessed 4,028 times by 2,970 users. Additionally, there have been 3,266 total downloads on the page during this period. This represents an encouraging initial step, as the document has been consulted and downloaded. The next step is to identify a method of encouraging oncology healthcare providers to provide feedback on the use of the MOATT in practice and research.

## Conclusion

In conclusion, the MOATT remains an important resource in supporting patients on oral anti-cancer agents, yet its underutilization in clinical practice presents a significant barrier to its full potential. Despite the evidence supporting its benefits, the limited documentation and inconsistent application highlight the need for a more strategic approach to implementation. To truly integrate MOATT into oncology care, it is essential to leverage implementation science, addressing the contextual and organizational factors that impact its use. By doing so, we can ensure that this tool not only reaches more patients but also enhances adherence and outcomes in a sustainable manner. The path forward requires a commitment to continuous feedback and adaptation, enabling the MOATT to evolve in alignment with the ever-changing landscape of cancer care.

## Data Availability

No datasets were generated or analysed during the current study.
